# Design, implementation, and evaluation of a software for unintentional injuries prevention in children 

**Published:** 2022-12

**Authors:** Zahra Tayebi, Seyede Soghra Taher Harikandee, Mohsen Lotfi

**Affiliations:** ^*a*^ Nursing Faculty, Alborz University of Medical Sciences, Karaj, Iran.; ^*b*^ Social Determinants of Health Research Center, Alborz University of Medical Sciences, Karaj, Iran.; ^*c*^ Takht-e Jamshid Hospital, Alborz University of Medical Sciences, Karaj, Iran.

## Abstract

**Background:**

Although unintentional injuries are preventable, political and social commitment is a prerequisite. Therefore, the volume of efforts leading to child injury prevention is still unacceptable. Technology-based interventions can provide parents with the required information whenever they need it. This study was carried out to design and assess injury prevention software for childcare providers.

**Methods:**

Educational content was provided by the researchers. The presented idea and plan were visualized as storyboards which were then converted into digital designs by Photoshop CS4. After designing and uploading the application in the BAZAR software (https://cafebazaar.ir/app/ir.ac.abzums?l=en), the evaluation was done through a qualitative study.

**Results:**

"News and Incidents," "Prevention," "Primary Intervention," and "Products" are the four main sections of the software. Two groups of children aged under one year old and one to five years old were considered when preparing the content. Most of the major unintentional injuries were included in the software (Poisoning, Falling, Burns, Suffocation, and Drowning). Items that needed to be improved were noted in the evaluation section, including the need for more visual symbols and the presence of important notifications.

**Conclusions:**

By using this software, costly booklets can be replaced, and more clients will be educated. The graphics and some contents of the software can be modified in order to be more attractive and enhance concept memorization.

**Keywords:**

Injury prevention, Drowning, Software, Technology

